# Supplementary material for: Comparative multiomics analysis of cell physiological state after culture in a basket bioreactor
Source: Sci Rep. 2022 Nov 23;12:20161. doi: 10.1038/s41598-022-24687-4 (PMC9686226; doi:10.1038/s41598-022-24687-4)
Supplement: Supplementary file 1 — Supplementary Information 1. [file 41598_2022_24687_MOESM1_ESM.zip › raw data/Transcriptomic raw data/6.Enrichment/2.KEGG/TE1vsTC1/GSEA/gsea_report_for_TE1_1656483128235.html]

Report for TE1 1656483128235 [GSEA]

| GS  follow link to MSigDB | GS DETAILS | SIZE | ES | NES | NOM p-val | FDR q-val | FWER p-val | RANK AT MAX | LEADING EDGE || 1 | CARBON\_METABOLISM(CSAB01200) | Details ... | 2 | 0.94 | 1.30 | 0.000 | 0.984 | 0.490 | 628 | tags=50%, list=3%, signal=51% |
| 2 | BIOSYNTHESIS\_OF\_AMINO\_ACIDS(CSAB01230) | Details ... | 2 | 0.94 | 1.30 | 0.000 | 0.492 | 0.490 | 628 | tags=50%, list=3%, signal=51% |
| 3 | CELL\_CYCLE(CSAB04110) | Details ... | 3 | 0.70 | 1.25 | 0.000 | 0.639 | 0.816 | 2496 | tags=67%, list=11%, signal=75% |
| 4 | PI3K\_AKT\_SIGNALING\_PATHWAY(CSAB04151) | Details ... | 3 | 0.70 | 1.25 | 0.000 | 0.479 | 0.816 | 2496 | tags=67%, list=11%, signal=75% |
| 5 | PROTEIN\_PROCESSING\_IN\_ENDOPLASMIC\_RETICULUM(CSAB04141) | Details ... | 3 | 0.49 | 1.25 | 0.000 | 0.417 | 0.816 | 1974 | tags=33%, list=9%, signal=37% |
| 6 | METABOLIC\_PATHWAYS(CSAB01100) | Details ... | 12 | 0.57 | 1.25 | 0.000 | 0.375 | 0.816 | 628 | tags=8%, list=3%, signal=9% |
| 7 | HUMAN\_PAPILLOMAVIRUS\_INFECTION(CSAB05165) | Details ... | 4 | 0.61 | 1.23 | 0.000 | 0.427 | 0.816 | 2496 | tags=50%, list=11%, signal=56% |
| 8 | SALMONELLA\_INFECTION(CSAB05132) | Details ... | 2 | 0.60 | 1.14 | 0.248 | 0.919 | 1.000 | 8883 | tags=100%, list=40%, signal=166% |
| 9 | BIOSYNTHESIS\_OF\_COFACTORS(CSAB01240) | Details ... | 3 | 0.87 | 1.14 | 0.000 | 0.836 | 1.000 | 628 | tags=33%, list=3%, signal=34% |
| 10 | VIRAL\_CARCINOGENESIS(CSAB05203) | Details ... | 5 | 0.53 | 1.11 | 0.197 | 0.973 | 1.000 | 2496 | tags=40%, list=11%, signal=45% |
| 11 | FOXO\_SIGNALING\_PATHWAY(CSAB04068) | Details ... | 4 | 0.55 | 1.09 | 0.334 | 0.989 | 1.000 | 2496 | tags=50%, list=11%, signal=56% |
| 12 | CELLULAR\_SENESCENCE(CSAB04218) | Details ... | 4 | 0.55 | 1.09 | 0.334 | 0.907 | 1.000 | 2496 | tags=50%, list=11%, signal=56% |
| 13 | GLYCINE\_\_SERINE\_AND\_THREONINE\_METABOLISM(CSAB00260) | Details ... | 1 | 0.97 | 1.08 | 0.000 | 0.975 | 1.000 | 628 | tags=100%, list=3%, signal=103% |
| 14 | CYSTEINE\_AND\_METHIONINE\_METABOLISM(CSAB00270) | Details ... | 1 | 0.97 | 1.08 | 0.000 | 0.905 | 1.000 | 628 | tags=100%, list=3%, signal=103% |
| 15 | VITAMIN\_B6\_METABOLISM(CSAB00750) | Details ... | 1 | 0.97 | 1.08 | 0.000 | 0.845 | 1.000 | 628 | tags=100%, list=3%, signal=103% |
| 16 | ENDOCYTOSIS(CSAB04144) | Details ... | 3 | 0.61 | 1.08 | 0.000 | 0.803 | 1.000 | 8751 | tags=100%, list=39%, signal=164% |
| 17 | UBIQUITIN\_MEDIATED\_PROTEOLYSIS(CSAB04120) | Details ... | 3 | 0.53 | 1.03 | 0.414 | 0.883 | 1.000 | 1974 | tags=33%, list=9%, signal=37% |
| 18 | MAPK\_SIGNALING\_PATHWAY(CSAB04010) | Details ... | 1 | 0.66 | 0.99 | 0.333 | 1.000 | 1.000 | 7523 | tags=100%, list=34%, signal=151% |
| 19 | CYTOKINE\_CYTOKINE\_RECEPTOR\_INTERACTION(CSAB04060) | Details ... | 1 | 0.66 | 0.99 | 0.333 | 1.000 | 1.000 | 7523 | tags=100%, list=34%, signal=151% |
| 20 | TGF\_BETA\_SIGNALING\_PATHWAY(CSAB04350) | Details ... | 1 | 0.66 | 0.99 | 0.333 | 1.000 | 1.000 | 7523 | tags=100%, list=34%, signal=151% |
| 21 | APELIN\_SIGNALING\_PATHWAY(CSAB04371) | Details ... | 1 | 0.66 | 0.99 | 0.333 | 1.000 | 1.000 | 7523 | tags=100%, list=34%, signal=151% |
| 22 | OSTEOCLAST\_DIFFERENTIATION(CSAB04380) | Details ... | 1 | 0.66 | 0.99 | 0.333 | 0.992 | 1.000 | 7523 | tags=100%, list=34%, signal=151% |
| 23 | ADHERENS\_JUNCTION(CSAB04520) | Details ... | 1 | 0.66 | 0.99 | 0.333 | 0.949 | 1.000 | 7523 | tags=100%, list=34%, signal=151% |
| 24 | TH17\_CELL\_DIFFERENTIATION(CSAB04659) | Details ... | 1 | 0.66 | 0.99 | 0.333 | 0.909 | 1.000 | 7523 | tags=100%, list=34%, signal=151% |
| 25 | RELAXIN\_SIGNALING\_PATHWAY(CSAB04926) | Details ... | 1 | 0.66 | 0.99 | 0.333 | 0.873 | 1.000 | 7523 | tags=100%, list=34%, signal=151% |
| 26 | AGE\_RAGE\_SIGNALING\_PATHWAY\_IN\_DIABETIC\_COMPLICATIONS(CSAB04933) | Details ... | 1 | 0.66 | 0.99 | 0.333 | 0.839 | 1.000 | 7523 | tags=100%, list=34%, signal=151% |
| 27 | CHAGAS\_DISEASE(CSAB05142) | Details ... | 1 | 0.66 | 0.99 | 0.333 | 0.808 | 1.000 | 7523 | tags=100%, list=34%, signal=151% |
| 28 | HUMAN\_T\_CELL\_LEUKEMIA\_VIRUS\_1\_INFECTION(CSAB05166) | Details ... | 1 | 0.66 | 0.99 | 0.333 | 0.779 | 1.000 | 7523 | tags=100%, list=34%, signal=151% |
| 29 | PATHWAYS\_IN\_CANCER(CSAB05200) | Details ... | 1 | 0.66 | 0.99 | 0.333 | 0.752 | 1.000 | 7523 | tags=100%, list=34%, signal=151% |
| 30 | COLORECTAL\_CANCER(CSAB05210) | Details ... | 1 | 0.66 | 0.99 | 0.333 | 0.727 | 1.000 | 7523 | tags=100%, list=34%, signal=151% |
| 31 | PANCREATIC\_CANCER(CSAB05212) | Details ... | 1 | 0.66 | 0.99 | 0.333 | 0.704 | 1.000 | 7523 | tags=100%, list=34%, signal=151% |
| 32 | CHRONIC\_MYELOID\_LEUKEMIA(CSAB05220) | Details ... | 1 | 0.66 | 0.99 | 0.333 | 0.682 | 1.000 | 7523 | tags=100%, list=34%, signal=151% |
| 33 | HEPATOCELLULAR\_CARCINOMA(CSAB05225) | Details ... | 1 | 0.66 | 0.99 | 0.333 | 0.661 | 1.000 | 7523 | tags=100%, list=34%, signal=151% |
| 34 | GASTRIC\_CANCER(CSAB05226) | Details ... | 1 | 0.66 | 0.99 | 0.333 | 0.642 | 1.000 | 7523 | tags=100%, list=34%, signal=151% |
| 35 | PEROXISOME(CSAB04146) | Details ... | 1 | 0.81 | 0.96 | 0.658 | 0.733 | 1.000 | 4305 | tags=100%, list=19%, signal=124% |
| 36 | HERPES\_SIMPLEX\_VIRUS\_1\_INFECTION(CSAB05168) | Details ... | 3 | 0.49 | 0.96 | 0.329 | 0.717 | 1.000 | 1371 | tags=33%, list=6%, signal=36% |
| 37 | HIPPO\_SIGNALING\_PATHWAY(CSAB04390) | Details ... | 2 | 0.47 | 0.76 | 0.801 | 0.952 | 1.000 | 7523 | tags=50%, list=34%, signal=75% |
| 38 | CELL\_ADHESION\_MOLECULES(CSAB04514) | Details ... | 2 | 0.54 | 0.75 | 0.662 | 0.931 | 1.000 | 2682 | tags=50%, list=12%, signal=57% |
| 39 | UBIQUINONE\_AND\_OTHER\_TERPENOID\_QUINONE\_BIOSYNTHESIS(CSAB00130) | Details ... | 1 | 0.57 | 0.72 | 0.658 | 0.917 | 1.000 | 9582 | tags=100%, list=43%, signal=175% |
Table: Gene sets enriched in phenotype **TE1 (2 samples) **[plain text format]****
